# Enhanced Virulence of *Chlamydia muridarum* Respiratory Infections in the Absence of TLR2 Activation

**DOI:** 10.1371/journal.pone.0020846

**Published:** 2011-06-14

**Authors:** Xianbao He, Anjali Nair, Samrawit Mekasha, Joseph Alroy, Catherine M. O'Connell, Robin R. Ingalls

**Affiliations:** 1 Section of Infectious Diseases, Boston Medical Center, Boston University School of Medicine, Boston, Massachusetts, United States of America; 2 Department of Pathology, Tufts University School of Medicine, Cummings School of Veterinary Medicine, and Tufts Medical Center, Boston, Massachusetts, United States of America; 3 Department of Pediatrics, Children's Hospital of Pittsburgh of UPMC, Pittsburgh, Pennsylvania, United States of America; University of California Los Angeles, United States of America

## Abstract

*Chlamydia trachomatis* is a common sexually transmitted pathogen and is associated with infant pneumonia. Data from the female mouse model of genital tract chlamydia infection suggests a requirement for TLR2-dependent signaling in the induction of inflammation and oviduct pathology. We hypothesized that the role of TLR2 in moderating mucosal inflammation is site specific. In order to investigate this, we infected mice via the intranasal route with *C. muridarum* and observed that in the absence of TLR2 activation, mice had more severe disease, higher lung cytokine levels, and an exaggerated influx of neutrophils and T-cells into the lungs. This could not be explained by impaired bacterial clearance as TLR2-deficient mice cleared the infection similar to controls. These data suggest that TLR2 has an anti-inflammatory function in the lung during Chlamydia infection, and that the role of TLR2 in mucosal inflammation varies at different mucosal surfaces.

## Introduction

*Chlamydia trachomatis* is the most common bacterial sexually transmitted pathogen worldwide [1], and the etiologic agent of blinding trachoma [2]. Complications of infections in women include the development of pelvic inflammatory disease, which can lead to tubal infertility and chronic pelvic pain. Infants born to women with active cervical chlamydia infection are at risk of developing conjunctivitis and pneumonia [3], [4]. While the induction of an inflammatory immune response is clearly essential for the survival of a host following an infectious challenge, it is also true that an exaggerated inflammatory response can be detrimental, as in the case of severe sepsis and some autoimmune chronic inflammatory states. Likewise, the pathology associated with genital chlamydia infection and ocular trachoma is thought to result from bystander injury during an aggressive inflammatory response. Thus, proinflammatory pathways must be tightly regulated.

The interaction of Chlamydia with innate immune receptors expressed at mucosal surfaces likely triggers the initial wave of proinflammatory mediators, which in turn recruit additional inflammatory cells, leading to further modulation of the immune response. The inflammatory pathways utilized by Chlamydia appear to primarily involve TLR2, and a number of studies have shown a dependence on expression of TLR2 in cellular activation by *C. trachomatis in vitro* [5], [6], [7], [8]. However, the TLR2 ligand remains unidentified, although lipoproteins expressed in the outer membrane of the organism are likely candidates [5]. Chlamydia species also express lipopolysaccharide (LPS) in the outer membrane, a recognized ligand for TLR4. However, unlike enteric LPS preparations, which are some of the most potent inducers of inflammation known, chlamydia LPS is of low endotoxic activity, and appears to be more dependent on membrane CD14 than has been reported for other species of LPS [9], [10], [11]. In addition, Chlamydia has been reported to activate type I interferon signaling in a TLR2- independent manner, possibly via a yet unidentified cytosolic DNA sensor [12].

Several small animal models exist for studying the pathogenesis of chlamydia infections. Significant data has been gathered from the female mouse model of ascending genital tract infection that closely parallels *C. trachomatis* infections and pelvic inflammatory disease in humans. When inoculated intravaginally into mice, *C. muridarum*, the mouse biovar of *C. trachomatis* (formerly known as MoPn), establishes an active infection of the endocervix, then ascends to the upper genital tract where it infects the oviducts. Consequences of oviduct infection in the mouse include dilatation (hydrosalpinx) and infertility [13], [14], [15]. In addition to being used in a model for genital tract infections, *C. muridarum* is a respiratory pathogen in mice, and produces pneumonia following intranasal inoculation [16]. *In vivo* data using TLR2-deficient mice [6] demonstrates an association between TLR2 expression and the development of inflammation and oviduct pathology following genital tract challenge with *C. muridarum* in the mouse model. While the bacterial burden and course of infection in the genital tract is unchanged in the absence of TLR2, expression of proinflammatory cytokines and chemokines in the genital tract is reduced, and upper tract pathology is diminished or absent. Similar results were obtained when mice were challenged with plasmid-cured mutants of *C. muridarum* that fail to activate TLR2 [17]. No studies to date have monitored the course of infection in the absence of TLR2 following respiratory challenge with *C. muridarum*.

We hypothesized that the role of TLR2 in regulating mucosal inflammation varies depending on the specific site. In order to determine the role of TLR2 signaling at the respiratory mucosal surface, we infected wild type and TLR2-deficient mice with *C. muridarum*. We found that while both mouse strains cleared the infection over time in a similar manner, the disease was more severe in TLR2-deficient mice, and these mice developed significantly higher lung cytokine levels with exaggerated neutrophil and T-cell influx in the lungs. Similar results were obtained when wild type mice were infected with plasmid-cured mutants of *C. muridarum* that fail to activate TLR2. Thus, our data suggests that TLR2 plays an important anti-inflammatory role in the lung during respiratory infection with Chlamydia.

## Materials and Methods

### Reagents

LPS (*Escherichia coli* serotype O111∶B4) was purchased from List Biological Laboratories, INC (Campbell, CA); RPMI-1640 was purchased from BioWhittaker® (Lonza Walkersville, MD). Fetal Bovine Serum (FBS) was from Hyclone (Logan, Utah). Renografin-60 was purchased from Bracco Diagnostics Inc. (Princeton, NJ).

### Bacterial preparation

*Chlamydia muridarum* strains Nigg (wild type) and plasmid-cured strains CM 972 and CM 3.1 have been previously described [8], [17], [18]. Chlamydiae were propagated in L929 fibroblasts growing in RPMI medium supplemented with 10% FBS at 37°C in a 5% CO_2_ environment. Following 42 to 45 hours of infection, cells were harvested, disrupted by glass beads or sonication (Sonicator 4000, Misonix Sonicators, Newtown, CT), and chlamydiae were separated from cell debris by ultracentrifugation through 32% Renografin. Chlamydial EBs were further purified by ultracentrifugation on a discontinuous Renografin gradient. After washing twice, the EB pellets were suspended in SPG (sucrose–phosphate–glutamate buffer, pH 7.2) and stored at −80°C prior to use. Bacterial titers were calculated as inclusion forming units (IFU) per ml. The stocks of *C. muridarum* strains used in this study were negative for *Mycoplasma* sp. by PCR [19].

### Mice

C57BL/6J mice were purchased from Jackson Laboratory (Bar Harbor, ME). TLR2-deficient mice generated by targeted deletion of mouse *tlr2* have been previously published [20] and were used with permission from Dr. Shizuo Akira. A colony of TLR2-deficient mice, back bred at least 10 generations on the C57BL/6 background, was maintained in our animal facility for use in these studies. All animals were housed in groups of 3–5 mice per cage in a controlled environment (temperature 20–22°C; 12∶12 hours light∶dark cycle), given free access to food and water, and maintained under the supervision of veterinary staff from the Laboratory Animal Science Center at Boston University Medical Center. All experimental procedures were carried out with approval from the Institutional Animal Care and Use Committee and the Institutional Biosafety Committee at Boston University Medical Center (Animal Welfare Assurance number A-3316-01).

### Preparation of alveolar macrophages and bone marrow derived macrophages

Alveolar macrophages (AM) were prepared from C57BL/6 and TLR2-deficient mice by bronchoalveolar lavage (BAL). Briefly, groups of 6–8 mice aged 6 to 8 weeks were euthanized by CO_2_ inhalation and the intact lungs lavaged with ∼1 ml of Hank's Balanced Salt Solution (HBSS). Collected AM were pooled and washed with PBS, then plated at 1.5×10^5^ cells/well on 48-well plates. Bone marrow derived macrophages (BMDM) were prepared from mice as follows. The bone marrows of femurs and tibiae from mice aged 6–8 weeks were flushed with RPMI1640 supplemented 10% FBS, 20 µg/ml gentamicin. The recovered cells were cultured in RPMI 1640 supplemented with 10% FBS, 20 µg/ml gentamicin, with 20–30% (v/v) of L929 conditioned medium (containing M-CSF) and incubated at 37°C, 5% CO_2_ for 7–9 days to facilitate macrophage differentiation before being infected with *C. muridarum*. Cells were inoculated with *C. muridarum* as described below.

### *In vitro* infection of cells with *C. muridarum*

Macrophages were seeded on tissue culture plates and rested overnight. The following day cells were inoculated with *C. muridarum* at the indicated multiplicity of infection (MOI). The plate was then centrifuged at 3000 rpm, 35°C for 1 h; this point was then considered as the 0 h time point. The inoculated macrophages were then incubated at 37°C, 5% CO_2_. At the designated time points, supernatant was collected and cells were washed twice with PBS, and then detached in 100 µl SPG and stored at −80°C for quantitative chlamydial culture.

### Murine intranasal infection model

Groups of 6–8 male or female mice aged 6 to 8 weeks were inoculated intranasally under light anesthesia using ketamine/xylazine mix (60–100/5–10 mg/kg i.p.). All infected mice received 20 µl bacterial suspension in phosphate buffered saline (PBS) containing 5×10^3^ IFU gradient purified chlamydiae unless otherwise noted in the text; mock infected mice received 20 µl of SPG diluted in PBS. Mice were weighed daily and observed for signs of distress that would require early euthanasia. At the indicated time points, mice were euthanized by CO_2_ inhalation. Lung tissue was process in one of two ways. For lung cytokines, flow cytometry and bacterial quantitation, lungs were homogenized in PBS using a Medimachine System (BD Biosciences, San Jose, CA). For histopathology and immunohistochemistry, lungs were inflated with 10% neutral formalin via the trachea, removed *en bloc* for further formalin fixation, and embedded in paraffin. Spleen homogenates were prepared in a similar fashion. All experiments involving mice were carried out with approval from the Institutional Biosafety Committee and the Institutional Animal Care and Use Committees at Boston University Medical Center. All *in vivo* experiments were repeated two to three times, as noted in the text.

### Quantification of *C. muridarum*

Bacterial load was determined by both quantitative culture and quantitative PCR. For culture of tissue, 150 µl of SPG was added to 50 µl of tissue homogenate, mixed, and briefly spun to pellet tissue debris. Serial dilutions were inoculated in duplicate onto L929 fibroblasts seeded to confluence in a 96-well plate. After incubation for 24–35 h at 37°C, 5% CO_2_, the cells were fixed with ice-cold methanol. Chlamydial inclusions in infected cells were detected using a Chlamydia-specific LPS monoclonal antibody (gift of Dr. You-Xun Zhang, Boston Medical Center), followed by FITC-conjugated secondary antibody; cells were counter stained with Evans blue (Sigma). The inclusions were counted under fluorescent microscopy, and calculated as the number of IFU per well/per lung.

Quantitative PCR was carried out from the same homogenates as follows: Genomic DNA was extracted from 5 µl of sample by addition to 195 µl of QuickExtract (Epicentre; Madison WI) and processed according to the manufacturer's instructions. The sample extracts were further diluted 1∶100 before being assayed via quantitative RT-PCR using primers directed against the 16S gene [21]. Genome equivalents per sample were extrapolated from a standard curve with adjustment for the presence of two copies of 16S on the *C. muridarum* genome.

### Detection of cytokines and chemokines

Lung homogenates and cell culture supernatants were assayed for cytokines and chemokines by individual ELISA and/or Milliplex 22-plex multiplex assay (Millipore Corp.; Billerica, MA), according to the manufacturer's instructions. ELISA kits for mouse IP-10 and TNF-α were purchased from R&D Systems (Minneapolis, MN) and eBioscience (San Diego, CA), respectively. ELISA plates were read in an ELx800 Universal Microplate Reader (BIO-TEX Instrument Inc.) and multiplex assays were run on the LiquiChip 200 Workstation (Qiagen, Valencia, CA). Each sample was assayed in triplicate (single ELISA) or duplicate (Multiplex), and *p*-values were calculated using an unpaired *t*-test. All *in vitro* studies were repeated at least three times.

### Flow cytometric analysis

Lung homogenates were digested in HEPES buffer (10 ml HEPES pH7.4, 150 mM NaCl, 5 mM KCl, 1 mM MgCl_2_, 1.8 mM CaCl_2_), containing collagenase D and DNase I at room temperature for 15 minutes and homogenized using a Medimachine System. A single-cell suspension was obtained by filtering the cells through a 70 µm mesh cell strainer. After lysis of the erythrocytes using RBC Lysis Buffer (eBioscience), cells were blocked using 1 mg goat IgG per ml, and incubated on ice with antibody at a final concentration of 0.5 µg/ml in phosphate buffered saline containing 1% fetal bovine serum for 30 minutes. The following directly conjugated fluorescent Abs were used for FACS analysis in these studies: F4/80-PE-Cy5 (clone BM8, from eBioscience) for the detection of macrophages; Ly-6G (Gr-1)-PE-Cy5 (clone RB6.8C5, from eBioscience) for the detection of neutrophils; CD19-PE (clone 6D5) and CD3-RPE (clone C363.29B; both from Southern Biotech, Birmingham, AL) for detection of B-cells and T-cells, respectively. Labeled cells were analyzed by flow cytometry using a fluorescence-activated cell sorter (FACScan) microfluorimeter (BD Biosciences) and analyzed using FlowJo software (Ashland, OR).

### Histopathology and immunohistochemical analysis

Following euthanasia, lungs were inflated with 10% neutral formalin via the trachea, removed *en bloc* for further formalin fixation, and embedded in paraffin. Lungs were cut completely in 7–8-µm sections, and every 10th section was stained with hematoxylin and eosin (H&E). For quantification of PMNs in lungs, chloroacetate esterase (CAE) stain was performed and PMNs were counted using ImageJ software. At least 10 fields were randomly reviewed from each lung. Immunohistochemistry was carried out on deparaffinized tissue that was stained with monoclonal antibodies recognizing mouse CD19 (clone MRQ-36; Cell Marque Corporation) vimentin (clone V9; Ventana), or their respective isotype controls, followed by an appropriate secondary antibody with horseradish peroxidase for detection. All slides were reviewed by a veterinary pathologist who was blinded as to the experimental design.

### Statistics

Significant differences for the *in vivo* studies were determined as follows. Statistical comparisons between the mouse strains for weight loss over the course of infection were made by a two-factor (days and mouse strain) repeated measures ANOVA with post hoc Bonferroni test. Statistical comparisons were made between murine strains for infectious burden at the time of euthanasia using a Mann-Whitney U test. Significant differences for the *in vitro* studies were determined using a two-tailed *t*-test. GraphPad Prism software was used for all statistical analyses.

## Results

### TLR2 expression is required for the induction of some inflammatory cytokines by macrophages *in vitro* in response to *C. muridarum* infection

**Figure 1:**
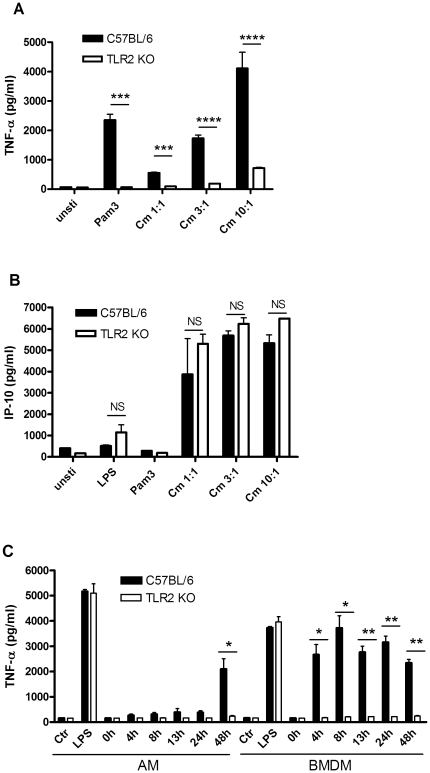
Cytokine induction in wild type vs. TLR2-deficient macrophages. (A) BMDM from C57BL/6 or TLR2 KO mice on the same background were left untreated, or infected with the indicated MOI of *C. muridarum* Nigg (Cm). Synthetic lipopeptide Pam_3_Cys-Ser-Lys_4_ (Pam3; 100 ng/ml) or LPS was used as a control. The supernatants were harvested at 24 hr post treatment and assayed for TNF-α and IP-10 by ELISA. The values showed are the mean ± SEM from triplicate samples. (B) BMDM and alveolar macrophages (AM) from C57BL/6 or TLR2 KO mice on the same background were infected with *C. muridarum* Nigg at MOI of 3∶1. LPS (100 ng/ml) was used as control. The supernatants were harvested at the indicated time points and assayed for TNF-α by ELISA. Significance was calculated as follows using a two-tailed *t*-test: *, p≤0.05; **, p≤0.01; ***, p≤0.001; ****, p≤0.0001.

Macrophages play a key role in the induction of a variety of proinflammatory cytokines, and they are rapidly recruited to mucosal surfaces along with other professional immune cells during infectious challenges. We first examined bone marrow derived macrophages from C57BL/6 vs. TLR2-deficient mice on the same background for induction of various cytokines in response to productive infection with *C. muridarum*. We observed the induction of cytokines such as TNF-α was largely dependent on expression of TLR2 (**Fig. 1A**), while a subset of cytokines, including IP-10, were TLR2 independent (**Fig. 1B**). The presence of resident macrophages is a unique property of the lung. We compared alveolar macrophages (AM) prepared from the lungs of control and TLR2-deficient mice with BMDM derived from the same mice to determine if alveolar macrophages differed in their response to chlamydia infection. We found TLR2 expression was also required for the induction of TNF-α by AM, (**Fig. 1C**) although when compared with BMDM, the absolute concentration is lower and the peak is markedly delayed. Thus, we anticipated that *in vivo* cytokine induction following intranasal challenge would also be largely TLR2-dependent, as has been observed in the genital tract [6].

### Disease following intranasal challenge is more severe in TLR2-deficient mice

**Figure 2:**
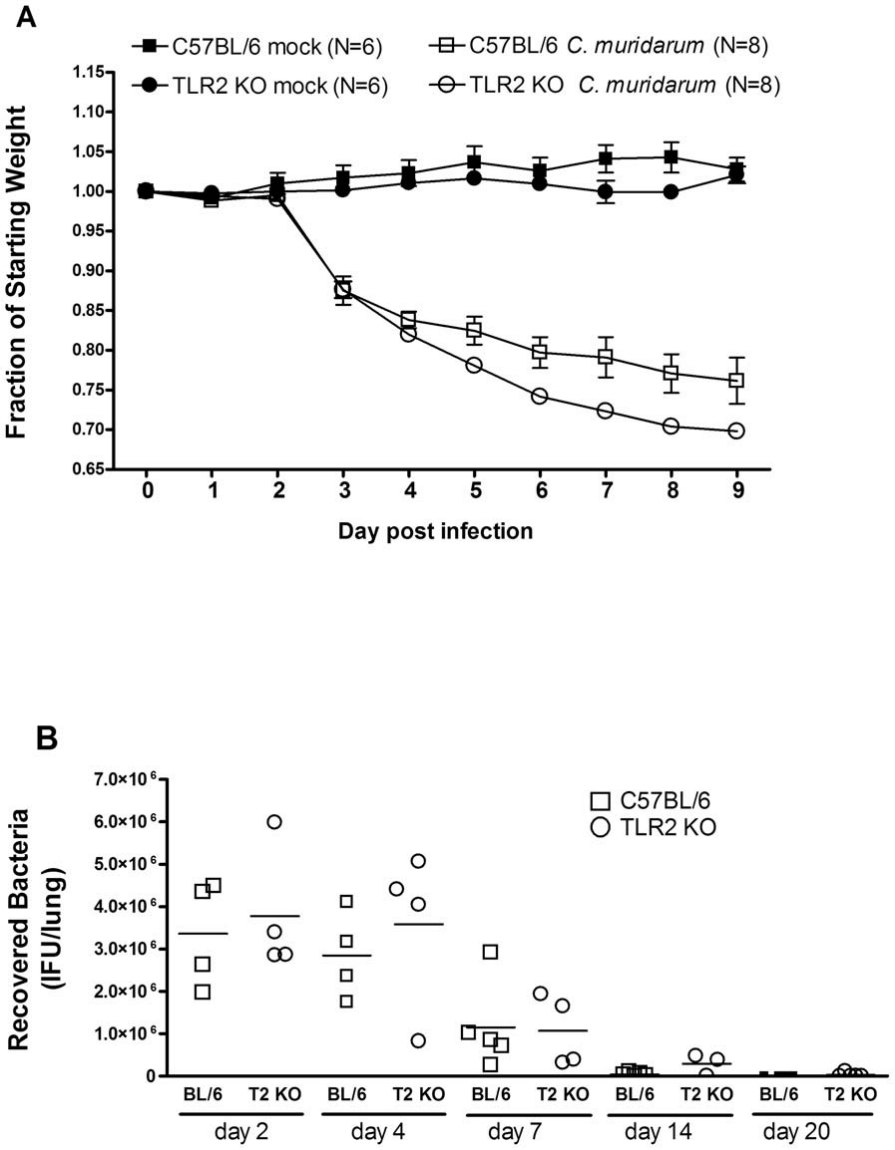
Weight change and bacterial clearance of *C. muridarum* infected wild type vs. TLR2-deficient mice. C57BL/6 or TLR2 KO mice on the same background were intranasally inoculated with PBS (mock; N = 6) or 2.5×10^4^ IFU/mouse of *C. muridarum* Nigg (N = 8), as described in the Methods. (A) Mice were weighed daily for 9 days post infection. Shown above is the weight change relative to the starting weight of each individual mouse (weight day X/weight day 0). Significance is as follows: p = 0.0474 infected BL/6 vs infected TLR2 KO over time, using RM ANOVA as described in the methods using a two-factor RM ANOVA with post hoc test. No significant difference in weight was observed between uninfected strains. (B) Mice were infected with 5×10^3^ IFU *C. muridarum* Nigg, and were euthanized on day 2, 4, 7, 14 and day 20. Quantitative bacterial cultures were determined from whole lung homogenates, as described in the Methods. Quantitative culture data was confirmed by quantitative PCR (data not shown). Statistical analysis of the infectious burden over time using a Mann-Whitney U test was not significant. This figure is representative of three independent experiments performed.

Intranasal infection of mice with *C. muridarum* is a well-established model for chlamydia-induced pneumonia. Previous studies have demonstrated that immune competent mice have peak bacterial burden at around 7 days post infection followed by bacterial clearance, and that IFN-γ and IL-12 are required for resolution of infection and protective immunity [22], [23]. To determine the role of TLR2 expression during respiratory infection with *C. muridarum*, we infected C57BL/6 and TLR2-deficient mice on the same background intranasally with a sublethal dose of bacteria (5×10^3^ IFU per mouse), and monitored for weight loss and survival. All infected mice, regardless of TLR2 expression, survived intranasal challenge, and while there was a trend towards exaggerated weight loss in the TLR2 deficient mice, it did not reach statistical significance (data not shown). However, when mice were challenged with a high inoculum of bacteria (2.5×10^4^ IFU per mouse), TLR2-deficient mice did show more exaggerated body weight loss over time compared to control C57BL/6 mice, particularly during the latter half of the course of infection, between days 5–9, suggesting more severe disease (**Fig. 2A**). Despite having more severe disease, we observed no difference in the peak bacterial burden or duration of infection between the two mouse strains regardless of the inoculum size (**Fig. 2B** and data not shown). The bacterial burden for infected C57BL/6 and TLR2-deficient mice peaked between days 2–4, and while there was a trend towards higher bacterial burden at day 4 in the TL2-deficient mice, it did not reach statistical significance.

### Infected TLR2-deficient mice develop higher inflammatory cytokine levels and more severe lung inflammation compared to wild-type mice when challenged via the intranasal route

**Figure 3:**
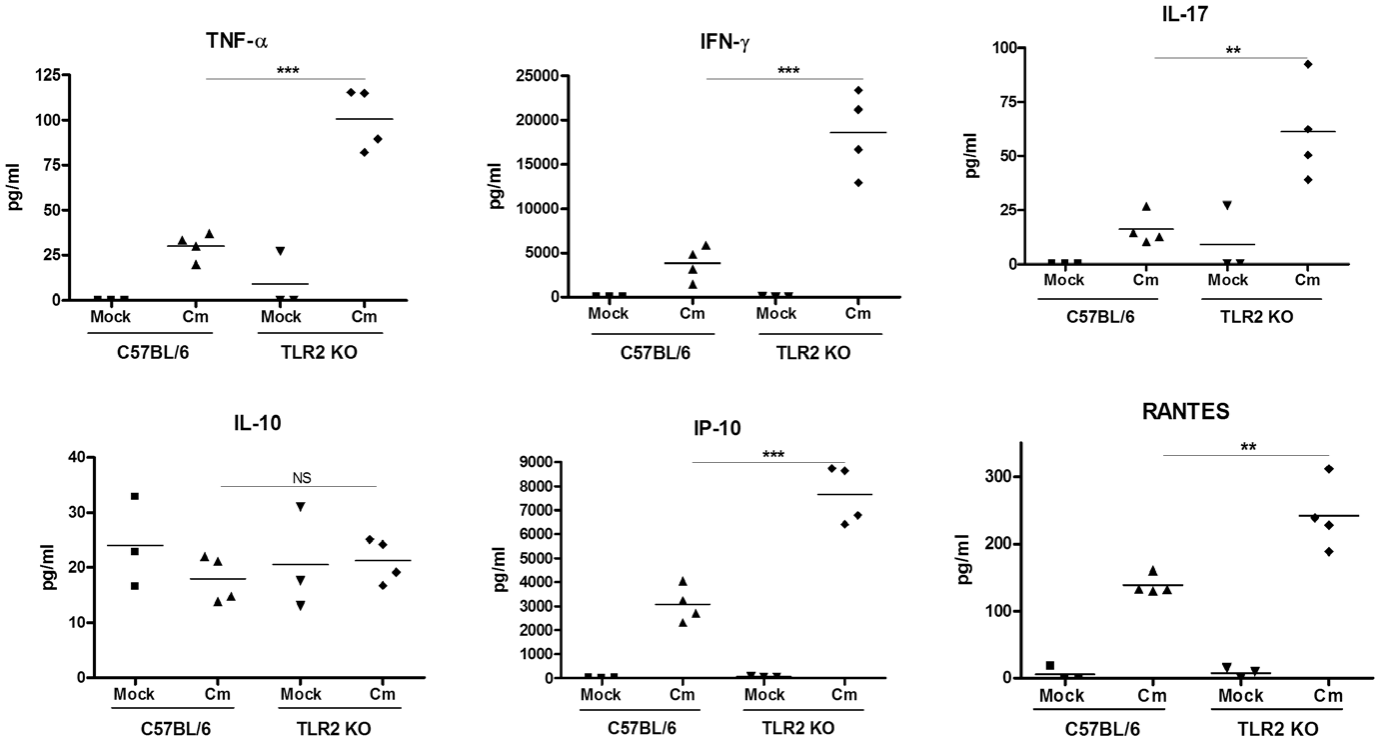
Intranasal infection of TLR2-deficient mice with *C. muridarum* induces more exaggerated lung inflammatory cytokine response compared to infected wild type mice. C57BL/6 or TLR2 KO mice on the same background were intranasally inoculated with PBS (mock) or 5×10^3^ IFU/mouse of *C. muridarum* Nigg, as described in the Methods. At seven days post infection, lung homogenates were assayed for a panel of 22 inflammatory cytokines and chemokines. Shown above are representative results for 6 of the cytokines assayed. Each data point represents one mouse, and the horizontal bar represents the mean. Significance was calculated as follows using a two-tailed *t*-test: **, p≤0.01 and ***, p≤0.001 for the infected C57BL/6 vs. infected TLR2-deficient mice. NS, not significant. This figure is representative of three independent experiments performed.

The trend towards increased weight loss following challenge that we observed in the TLR2-deficient mice suggested that they might have suffered more severe inflammation during the course of infection compared to the controls. Indeed, when we examined lung homogenates from infected mice at various points in time, we found that nearly all of the cytokines and chemokines tested, were increased in the infected TLR2-deficient mice relative to the control mice (**Fig. 3** and data not shown). This included classic proinflammatory cytokines, such as TNF-α, IL-6, and chemokines, such as MCP-1 and IP-10. Moreover, IFN-γ, IL-12 and IL-17, which have been shown to be required for bacterial clearance, were also elevated in the TLR2-deficient mice, possibly facilitating resolution of the infection in spite of more severe disease. Thus, in contrast to the *in vitro* infection, TLR2 expression was not required for the induction of cytokines *in vivo* following intranasal challenge.

**Figure 4:**
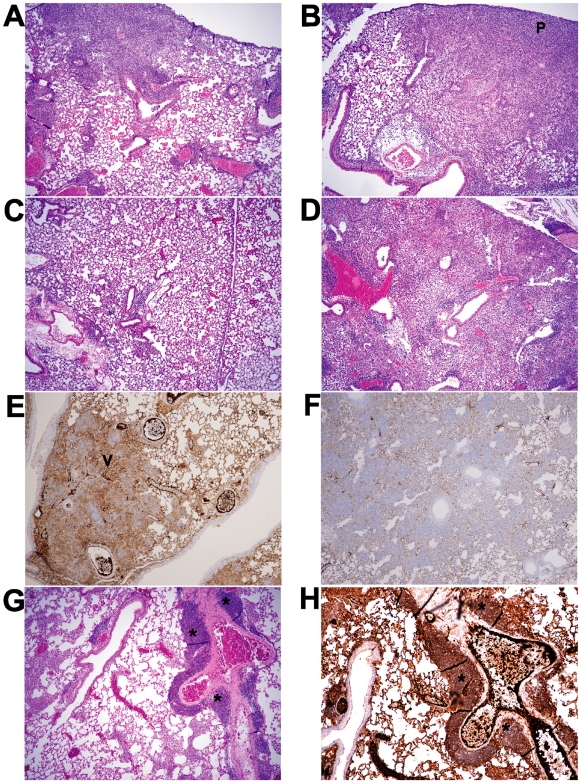
Intranasal infection with *C. muridarum* induces more extensive inflammatory changes in the lungs of TLR2-deficient mice compared to wild type mice. C57BL/6 or TLR2 KO mice on the same background were intranasally inoculated with PBS (mock) or 5×10^3^ IFU/mouse of *C. muridarum* Nigg. At the indicated time points, mice were euthanized and lungs were removed for tissue processing, as described in the Methods. A–D and G show routine H&E staining; P = dense PMN infiltrate, shown as deep purple-staining cells. E–F and H show vimentin and CD19 staining by immunohistochemistry, respectively. (A) C57BL/6, day 7; (B) TLR2-deficient, day 7; (C) C57BL/6, day 14; (D), TLR2-deficient, day 14. (E–F) Lungs from infected TLR2-deficient mice (F) fail to stain for vimentin (V) at 5 days post infection compared to C57BL/6 mice (E). (G–H) Lungs from infected TLR2-deficient mice show evidence of iBALT (*) at 7 days post infection when stained with H&E (G) or anti CD19 Ab to detect CD19-expressing B cells (H). Original magnification 40×. This figure is representative of three independent experiments performed.

**Figure 5:**
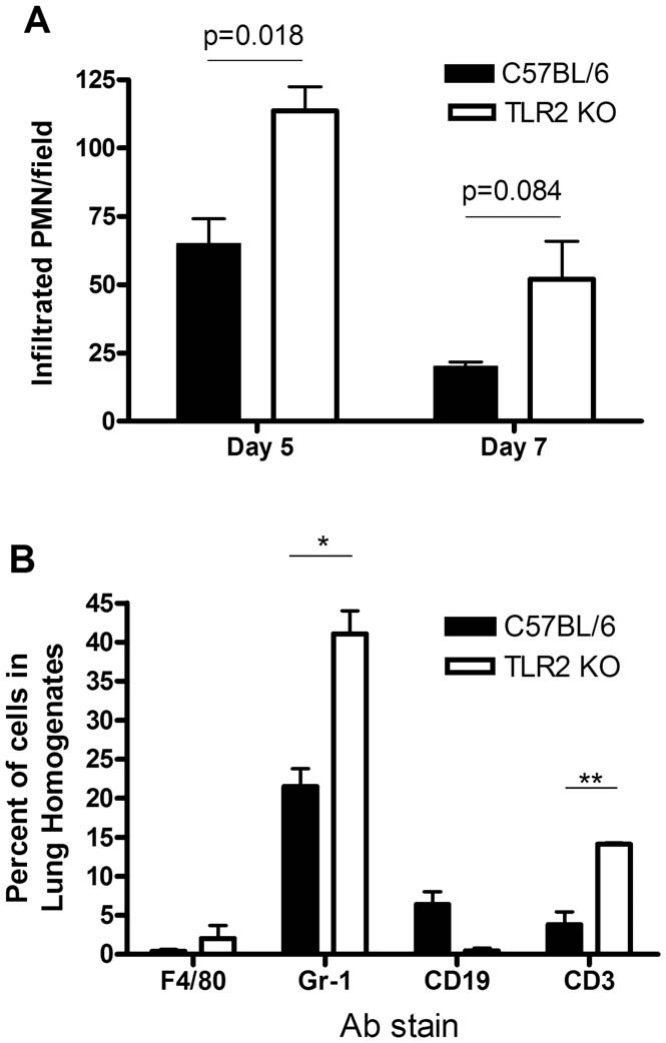
TLR2 deficiency leads to exaggerated PMN and T cell response in the lungs of C. muridarum infected mice. C57BL/6 or TLR2 KO mice on the same background were intranasally inoculated with 5×10^3^ IFU/mouse of *C. muridarum* Nigg. At the indicated day post infection, lungs from infected mice were processed to evaluate inflammatory cell infiltrates. (A) At day 5 and day 7 post-infection, lungs were fixed, sectioned and stained using CAE to quantify the PMN infiltration, as described in the Methods. Significance was calculated using an unpaired *t*-test, and the p-value is noted. (B) Single-cell suspensions of infected lungs at day 7 were prepared, stained, and analyzed by flow cytometry, as described in the Methods. F4/80-PE Cy5 was used as a marker for macrophages; Gr-1-PE Cy5 for neutrophils; CD19-PE for B lymphocytes and CD3-PE for T lymphocytes. The bar graph represents the percentage of positive cells from total lung cells, as determined by histogram data. Significance is as follows: *, p≤0.05; **, p≤0.01 for the infected C57BL/6 vs. infected TLR2-deficient mice using an unpaired *t*-test. This figure is representative of two independent experiments performed.

We next examined the lung tissue for evidence of inflammation. Both control and TLR2-deficient mice developed a patchy pneumonia, with inflammatory exudates visible in the alveolar space. As shown in figure 4, histological examination of lungs from infected mice by routine H&E staining at day 7 post infection revealed various size foci of pneumonia. In some of the foci the alveoli are packed with neutrophils, while in other foci the alveoli contained only few neutrophils but had proliferating fibroblasts. Overall, the TLR2-deficient mice had more severe neutrophilic infiltration while the wild type C57BL/6 mice displayed more prominent fibroblast proliferation, suggesting superior repair (**Fig. 4A, 4B**). PMN infiltration from infected mouse lungs was quantified, and is shown in **Fig. 5A**. Consistent with this observation, only lung tissue from the wild type mice stained for the intermediate filament vimentin at 5 days post infection (**Fig. 4E, 4F**), although by day 14 vimentin staining was similar in both mouse strains (data not shown). By 14 days post infection, inflammation was still visible in the TLR2-deficient mice while inflammation was largely resolved in the wild type mice (**Fig. 4C, 4D**). Moreover, in more than half of the TLR2-deficient mice, we observed prominent, well-developed bronchus associated lymphoid tissue (BALT) containing CD19-expressing B lymphocytes at 5 days post infection, consistent with inducible BALT (iBALT) (**Fig. 4G, 4H**). In contrast, while there were a few areas of poorly developed lymphoid aggregates seen in one of the C57BL/6 mice, it did not develop into the prominent BALT seen in the knockout mice (data not shown).

To confirm the observation that TLR2-deficient mice experienced more severe lung inflammation in response to their infection, we examined the recruitment of neutrophils and macrophages to the lung using flow cytometry. Lungs from infected mice were removed at 7 days post-infection, digested to obtain a single cell suspension, and stained for expression of GR1, a marker for neutrophils, F4/80, a marker for macrophages, CD19, a marker for B-cells, and CD3, a marker for T-cells. As shown in **Fig. 5B**, we found that TLR2-deficient mice had a significantly higher percent of infiltrating neutrophils and T-cells in the lungs when compared to control mice. Elevated levels of macrophages were also detected in these mice. Interestingly, B-cells appeared to be decreased in response to infection in the TLR2-deficient mice compared to wild type mice when analyzed by this method, although, as noted above, immunohistochemistry demonstrated the formation of B-cell aggregates into areas of BALT.

### Plasmid-cured strains of *C. muridarum* induce more inflammation compared to wild-type strain when inoculated via the intranasal route

**Figure 6:**
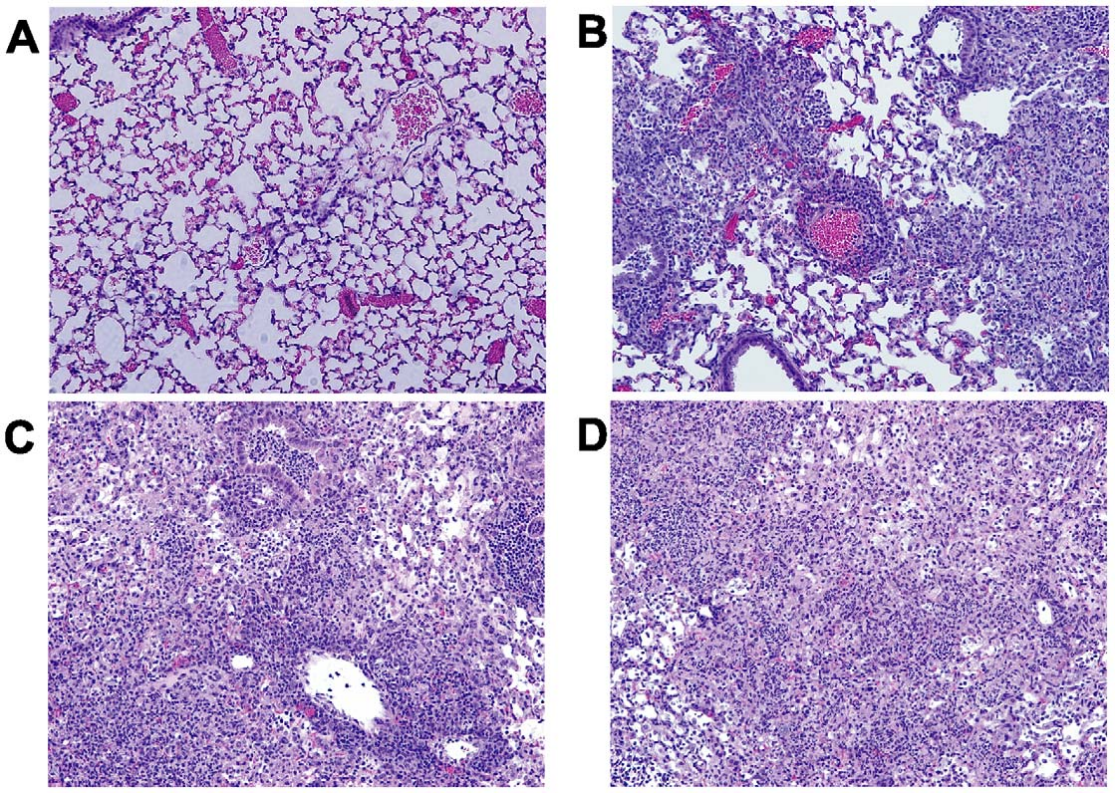
Intranasal infection with plasmid-cured strains of *C. muridarum* induces more extensive inflammatory changes in the lungs compared to wild type *C. muridarum* infected mice. Mice were inoculated via the intranasal route with *C. muridarum* Nigg or one of the plasmid-deficient mutant strains, CM972 or CM3.1, or mock infected with PBS, as described in the Methods. At seven days post infection, lungs were removed and the tissue processed for routine H&E staining. Shown above are representative histopathological images from (A) uninfected mice, and mice infected with (B) Nigg, (C) CM972, or (D) CM3.1. The dense, dark purple stain denotes the infiltrating PMNs, while the pale purple stain represents edema. Original magnification 100×. This figure is representative of two independent experiments performed.

**Figure 7:**
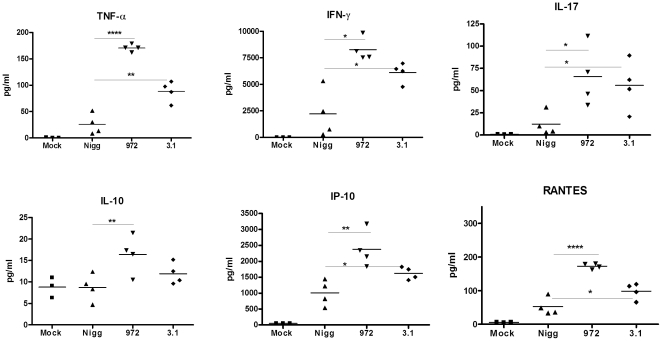
Intranasal infection with plasmid-cured strains of *C. muridarum* induces more exaggerated lung inflammatory cytokine response compared to wild type *C. muridarum* infected mice. Mice were inoculated via the intranasal route with *C. muridarum* Nigg or one of the plasmid-deficient mutant strains, CM972 or CM3.1, or mock infected with PBS, as described in the Methods. At seven days post infection, lung homogenates were assayed for a panel of 22 inflammatory cytokines and chemokines. Shown above are representative results for 6 of the cytokines assayed. Each data point represents one mouse, and the horizontal bar represents the mean. Significance is as follows: *, p≤0.05; **, p≤0.01; ***, p≤0.001; and ****, p≤0.001 for the wild type Nigg vs. plasmid mutant 972 or 3.1 infected mice using an unpaired *t*-test. This figure is representative of two independent experiments performed.

We previously described two plasmid-deficient *C. muridarum* Nigg strains, CM 972 and CM 3.1, which are impaired in their ability to activate TLR2 *in vitro* and in the murine genital tract infection model. Moreover, mice infected with these plasmid-cured strains fail to develop oviduct pathology *in vivo* [17]. Based on our data with the TLR2-deficient mice, we hypothesized that these mutants, although attenuated when inoculated in the genital tract, would display enhanced virulence when inoculated via the intranasal route. Indeed, we observed striking differences between mice infected with the wild type plasmid-containing Nigg strain compared with mice infected with either of the two plasmid-cured mutants. Mice infected with CM 972 or CM 3.1 strains were subjectively more ill-appearing compared to the Nigg-infected mice, and while there was a trend towards more exaggerated weight loss in the plasmid-cured infected mice, it did not reach statistical significance (data not shown). Upon histological examination, the lungs from all infected mice displayed evidence of pneumonia that was characterized by the presence of edema, with alveolar infiltration by neutrophils. However, mice infected with either CM 972 or CM 3.1 were grossly more edematous and necrotic when compared with the Nigg-infected mice (data not shown), and upon histologic examination they displayed significantly more interstitial edema and more extensive parenchymal involvement (**Fig. 6**). Furthermore, the CM972 and CM3.1 infected mice displayed significantly increased lung cytokines and chemokines compared to Nigg infected mice (**Fig. 7** and data not shown), although we observed no difference in bacterial clearance at 7 and 14 days post-infection (data not shown). These data resembled the results obtained from TLR2-deficient mice, and suggested that the inability of the plasmid-cured strains to activate TLR2-dependent signaling pathways led to an exaggerated inflammatory response in the lung. Thus, while the presence of the plasmid enhances chlamydial virulence in the genital tract it has the opposite effect in lung.

## Discussion

Mucosal surfaces are important sites of tolerance and infection in the respiratory, urogenital and gastrointestinal tracts and there is a growing interest in the development of vaccines capable of inducing both mucosal and systemic immunity in the fight against a variety of pathogens. However, immune defenses in these complex and unique mucosal compartments are not identical, and careful studies are needed to characterize the defense responses specific to each site. The murine pathogen, *C. muridarum*, provides an opportunity to compare innate immune defenses at two different mucosal sites because it infects both the genital tract and the lung of mice. Thus, inflammatory differences identified in our study between the lung and the genital tract may be ascribed to the site-specific immune response rather than the pathogen.

We observed that in the absence of TLR2 expression, mice had more severe disease when inoculated with *C. muridarum* via the intranasal route, with TLR2-deficient mice demonstrating more severe weight loss as well as a more exaggerated and prolonged inflammatory response in the lungs, as demonstrated by increased lung cytokines and cellular inflammatory cell infiltrate. The observation of enhanced induction of BALT in the lungs of TLR2-deficient mice is consistent with the overall exaggerated inflammatory response observed, as iBALT has been shown to develop in response to infection or chronic inflammation [reviewed in [24]]. Interestingly, mice infected with plasmid-cured strains of chlamydia that fail to activate TLR2 displayed a similarly exaggerated inflammatory response. Thus, TLR2 activation in the lung appears to be necessary for moderation of the inflammatory response both with regard to attenuating the induction of inflammatory cytokines and the inflammatory cellular response. This is the opposite of what has been observed in the genital tract where TLR2 activation is required for peak cytokine induction and the subsequent development of oviduct pathology [6], [17]. We do not believe that gender differences account for the difference in the host inflammatory response between mucosal sites, as we used both male and female mice in the lung infection model and we observed no significant difference in the clinical course and cytokine induction between male and female mice (data not shown). Instead, we believe that the role of TLR2 in driving inflammation and pathology at the two mucosal sites differs, likely driven by differences in the cellular composition and physiologic roles of the specific tissues.

The explanation for enhanced inflammation in the lung in response to chlamydial infection in the absence of TLR2 expression does not appear to be explained by impaired bacterial clearance or increased bacterial load because both wild type and TLR2-deficient mice displayed identical kinetics with regard to clearance of the infection. This observation was consistent with prior studies in the genital tract model indicating that that TLR2 expression is not necessary for clearance of the bacterial infection at that site as well [6]. However, it is possible that a transiently elevated bacterial load in the TLR2-deficient mice compared to the wild type mice that we could not detect might expose the tissue to other TLR ligands, thus driving inflammation. Others have reported an essential role for IL-17 and Th17 responses in the protection of mice against respiratory infection with *C. muridarum* [25], [26], [27]. However, we found no evidence of impaired IL-17 production in the TLR2-deficient mice that could explain the more severe infection. In fact, we observed an enhanced IL-17 response in the lung, which would be consistent with the exaggerated neutrophil response we also observed. Moreover, the induction of IL-10, an anti-inflammatory cytokine, was not impaired in the absence of TLR2 signaling.

One possible mechanism for our observation that TLR2-deficient mice developed exaggerated PMN response in the lung involves altered alveolar macrophage function. Alveolar macrophages have long been known to play an important role in the resolution of inflammation in the lung by virtue of their ability to phagocytose and clear apoptotic PMNs [28]. Given that Chlamydia-infected TLR2-deficient macrophages were impaired in their ability to secrete proinflammatory cytokines, they might also be impaired in their ability to clear PMNs from the lung, thus allowing inflammation to persist for longer. In addition, it was recently reported that engagement of TLR2 promotes the survival of regulatory T-cells [29], which might also be important in turning off inflammation in the lung. Regarding the observed differences between the lung and genital tract mucosal responses to the same pathogen, further studies are needed to determine whether differences in the expression of TLR2 on cell types that are specific to these two compartments might account for the differential role of TLR2 in inflammation in different tissues.

A protective role for TLRs in respiratory infections has been reported for a number of pathogens. For example, TLR4-deficient mice were found to be incapable of controlling Gram-negative respiratory infections secondary to *Klebsiella pneumoniae* [30], *Haemophilus influenzae* [31], and *Pasteurella pneumotropica* [32], [33]. Likewise, TLR2-deficient mice were recently reported to have more severe mycoplasma respiratory infections [34]. However, in all cases infected mice displayed enhanced bacterial growth in their lungs, which we did not observe in our Chlamydia-infected mice. This most likely reflects the ability of TLR2-deficient mice to mount an adequate IFN-γ response independent of TLR2 activation, and further suggests that the exaggerated inflammation is related to downstream anti-inflammatory pathways.

Finally it is worth comparing our results in the murine respiratory model for *C. muridarum* with the three published studies on the related pathogen, *C. pneumoniae*, all of which used TLR2-deficient mice. In the first report, the bacterial load in TLR2-deficient mice infected with *C. pneumoniae* was unchanged, and the inflammatory cytokine response in the lung was decreased [35]. Subsequently, two other groups reported similar findings with regard to bacterial clearance in TLR2-deficient mice and they reported no difference in lung inflammation [36], [37]. It is difficult to directly compare *C. muridarum* and *C. pneumoniae* models since they use related, but distinct, species of chlamydia. Moreover, *C. muridarum* is a natural respiratory pathogen of mice that likely evolved to infect this particular niche, and mice become symptomatic with a significantly lower inoculum (3 logs) compared to *C. pneumoniae*. The question remains as to which model more closely represents human respiratory disease, and it is likely that both models address different aspects of human infections from chlamydia.

TLRs and other innate immune receptors play a critical role in the innate immune response to pathogens, and the cooperation between multiple cell types and the panel of receptors they express maintains the balance between antimicrobial defenses and tissue homeostasis. Our observations lead us to conclude that the role of TLR2 in host defense is contextual, and that while TLR2 deficiency results in decreased virulence of infection in the genital tract, it has the opposite effect in the lung. Thus, the response to a pathogen at one mucosal surface may not necessarily predict the response at a different mucosal site, an important consideration for development of mucosal vaccines. A better understanding of the cellular interactions and molecular signaling pathways activated during infection at various mucosal surfaces is needed if the development of mucosal vaccines is to move forward.
